# Crop Management in Controlled Environment Agriculture (CEA) Systems Using Predictive Mathematical Models †

**DOI:** 10.3390/s20113110

**Published:** 2020-05-31

**Authors:** Chiara Amitrano, Giovanni Battista Chirico, Stefania De Pascale, Youssef Rouphael, Veronica De Micco

**Affiliations:** Department of Agricultural Sciences, University of Naples Federico II, 80055 Portici, Italy; chiara.amitrano@unina.it (C.A.); depascal@unina.it (S.D.P.); youssef.rouphael@unina.it (Y.R.)

**Keywords:** crop modelling, energy cascade model (MEC), *Lactuca sativa* L. var. *capitata*, controlled environment agriculture (CEA), precision horticulture

## Abstract

Proximal sensors in controlled environment agriculture (CEA) are used to monitor plant growth, yield, and water consumption with non-destructive technologies. Rapid and continuous monitoring of environmental and crop parameters may be used to develop mathematical models to predict crop response to microclimatic changes. Here, we applied the energy cascade model (MEC) on green- and red-leaf butterhead lettuce (*Lactuca sativa* L. var. *capitata*). We tooled up the model to describe the changing leaf functional efficiency during the growing period. We validated the model on an independent dataset with two different vapor pressure deficit (VPD) levels, corresponding to nominal (low VPD) and off-nominal (high VPD) conditions. Under low VPD, the modified model accurately predicted the transpiration rate (RMSE = 0.10 Lm^−2^), edible biomass (RMSE = 6.87 g m^−2^), net-photosynthesis (rBIAS = 34%), and stomatal conductance (rBIAS = 39%). Under high VPD, the model overestimated photosynthesis and stomatal conductance (rBIAS = 76–68%). This inconsistency is likely due to the empirical nature of the original model, which was designed for nominal conditions. Here, applications of the modified model are discussed, and possible improvements are suggested based on plant morpho-physiological changes occurring in sub-optimal scenarios.

## 1. Introduction

Environmental control is a key factor to increase plant productivity in controlled environment agriculture (CEA) [1]. Recently, increased interest has been directed towards plant production in closed facilities (e.g., plant factories, vertical farms, indoor-growing modules) [2,3,4]. However, with the introduction of advanced monitoring and control technologies, it becomes necessary to properly discern plant/microclimate interaction, modulate environmental parameters, and manage cultivation factors. Indeed, in protected agriculture, environmental factors and plant responses are strictly interconnected: alteration of the microclimate can induce modifications in plants (both at morphological and physiological levels), affecting plant behavior especially in terms of transpiration and CO_2_/O_2_ exchanges, which in turn re-modify the surrounding environment [5].

Recently, a technological “revolution” in agriculture is on-going, pushed by advances in sophisticated technologies such as robots, aerial/proximal sensors and more broadly by the internet of the things (IoT) [6]. This “revolution”, based on the automation of processes and the remote monitoring of systems, would allow the realization of smart farming, with a more lucrative, efficient, and sustainable production [7]. Remote sensing technologies allow the early monitoring of plant responses to environmental stresses such as: drought, salinity, and heat due to high temperature or excessive solar radiation. Currently, the remote sensing of plant physiological behavior is often based on reflectance indices, including photochemical reflectance index (PRI), normalized difference vegetation index (NDVI), leaf area index (LAI), and water index (WI) [8,9,10]. However, these indexes are mostly used in the field and their use for the remote monitoring of the photosynthetic process under controlled conditions is still limited. Indeed, in protected cultivations, crop monitoring mostly relies on sensors controlling environmental parameters, and on plant gas-exchange and fluorescence analyses [10].

As we progress in adopting technological advancement, issues related to sensor loss of control or breakage may be experienced. Such phenomena would be responsible for modifications occurring at the plant level during the cultivation cycle, such as alterations in plant growth, morphogenesis, and development. In this context, mathematical models, which mimic the behavior of real systems, can be adopted to monitor, simulate, predict, control, and facilitate the understanding of crop behavior in protected cultivation under both nominal (optimal) and off-nominal (sub-optimal) conditions [11]. Until now, most of the controlling tasks in CEA have been designed to maintain specific set-points, neglecting the effects of environmental perturbations on crops [12]. However, evaluating crop status especially under off-nominal conditions represents an added value to forecast possible growth reductions and prevent yield losses by a real-time fine-tuning of environmental control and water management (irrigation schedule), according to different plant phenological stages.

The aerial (moist/dry) control and, more specifically, the Vapor Pressure Deficit (VPD) regulation is listed amongst the main critical issues for crop production in CEA [13]. Indeed, VPD is one of the main drivers for plant transpiration: it affects crops during growth, changing plant morpho-physiological development, especially impacting water fluxes in the soil–plant–atmosphere continuum (SPAC), and thus the availability of water during the cultivation [14]. Given that plant transpiration is recognized to be a convenient indicator of its water status, real-time sensor information is a fundamental pre-requisite for the precision irrigation management of crops [15]. Furthermore, in small-indoor-growing modules, like those used for cultivation in space, the regulation of plant water fluxes can be easily disrupted due to difficulties in the control of VPD in limited volumes. Therefore, a proper irrigation management is even more recommended.

These difficulties in VPD control in protected cultivation can determine a rise in air temperature and consequently in evapotranspiration, which impact crop production, also resulting in decreasing stomatal conductance and photosynthetic rates [16]. Transpiration in plants is influenced by environmental conditions and regulated by stomatal opening/closing [17]. High VPD values (1.7–2 kPa) intensify plant physiological stress, especially under water shortage, by increasing plant water loss and decreasing carbon fixation, thus negatively influencing crop growth and productivity, which represents a major issue for production in CEA [18]. Furthermore, together with increasing VPD and water stress, ABA hormone tends to accumulate in leaves and its concentration is negatively correlated with the stomatal conductance, further exacerbating leaf transpiration [19].

Nowadays, there are numerous models which simulate different photosynthetic and plant productivity processes, often focusing on very specific aspects of plant physiology, such as: protection of photosynthetic apparatus through the non-photochemical quenching, mesophyll conductance to CO_2_, genotype-environment interactions [20,21,22,23]. Among these models, the energy cascade model (MEC) has already been tested to implement crop growth in small prototypes for bioregenerative life support systems (BLSSs) studies and in a lunar/Martian greenhouse [12,24]. This “explanatory” model is therefore considered suitable to predict both biomass, photosynthesis, transpiration and energy balance in closed systems. Moreover, such a model could easily be applied by stakeholders operating in controlled agriculture facilities for food production. Indeed, the model, requiring just a few environmental and cultivation parameters as inputs, can help forecasting changes happening during the cultivation after the modification of environmental factors, thus being possibly implemented as decision support system.

In the present study, we report an application of the MEC model on butterhead lettuce (*Lactuca sativa* L. var. *capitate*) cultivated under controlled conditions, also presenting a modification to the original model. More specifically, we introduced additional components to include the variation of canopy quantum yield of PSII (CQY) and carbon use efficiency (CUE) during leaf development. Such parameters, which represent respectively the moles of carbon fixed per mole of photons absorbed and the ratio of net carbon gain to gross carbon assimilation during growth, are critical to the model for the calculation of the net photosynthesis and the biomass production. Furthermore, we applied the modified version of the model on green- and red-leaf lettuce grown in a climatic chamber under two VPD scenarios, namely low and high VPD, corresponding to nominal and off-nominal conditions, respectively. This latter trial allowed the portrayal of the model parameters for nominal and off-nominal scenarios for green- and red-leaf plants, since non-identical behavior often occurs for different cultivars/varieties even under the same growth conditions. 

## 2. Materials and Methods

### 2.1. The Original MEC Model

The original energy cascade model was developed for wheat by Volk et al. [25] and then calibrated for other crops like lettuce, rice, soybean, sweet potato and tomato [26]. It is an explanatory model, composed by multivariate equations whose coefficients have been determined through curve fitting of experimental data [27]. The input variables of the first version of the model were the light intensity (photosynthetic photon flux density; PPFD) and the photoperiod. All the model outputs, which mostly concerned the biomass and the growth rate, were function of these two parameters. 

The original MEC model was based on an “energy cascade” with three fundamental steps:

(1) the absorption of PPFD by the canopy;
(2) the absorbed energy (A) used in the photosynthetic process to convert carbon into sucrose;
(3) the conversion of sucrose into biomass.

Such a simple model required only three crop parameters, namely (i) the time of canopy closure (t_A_), (ii) the time of senescence onset (t_Q_), and (iii) the time of harvesting (t_M_). Eventually, the model was modified to add the following climatic parameters: air temperature, relative humidity, carbon dioxide concentration, dark period, and plant density, in order to improve the accuracy and robustness of the model [16]. In 2012, Boscheri et al. [12] implemented a modified version of the MEC model, for a multi-crop Lunar greenhouse prototype. The version modified by Boscheri et al. [12] also included a crop transpiration component, used to predict water and plant nutrient consumption. 

The main model algorithm components were arranged according to twelve equations, sequentially computed at each time step to calculate the key variables as listed below.

The canopy quantum yield (CQY, mol^−1^) is defined by an empirical equation as function of the time t, expressed in days after emergence:

CQY = CQY_MAX_              for t ≤ t_Q_ CQY = CQY_MAX_ − (CQY_MAX_ − CQY_MIN_)(t − t_Q_)/(t_M_ − t_Q_) for t_Q_ < t ≤ t_M_ (1)

where CQY_MAX_ and CQY_MIN_ are crop-specific parameters, while t_Q_ and t_M_ are time of the onset of senescence and time of harvesting, respectively. 

The carbon use efficiency (CUE), is expressed similarly to CQY, according to the following relation:

CUE = CUE_MAX_              for t ≤ t_Q_ CUE = CUE_MAX_ − (CUE_MAX_ − CUE_MIN_) (t − t_Q_)/(t_M_ − t_Q_) for t_Q_ < t ≤ t_M_ (2)

where CUE_MAX_ and CUE_MIN_ are crop specific parameters, although CUE has been often assumed to be constant for most crops (i.e., CUE_MAX_ = CUE_MIN_), t_Q_ and t_M_ are time of the onset of senescence and time of harvesting, respectively.

The parameter A is the fraction of PPFD absorbed by the top of the canopy and is assumed to increase with time t according to a power law equation, up to a maximum value A_MAX_ at time t = t_A_, when canopy closure is established:

A *=* A_MAX_·(t/t_A_)^n^              for t ≤ t_A_ A = A_MAX_                     for t > t_A_ (3)

where t_A_ is the time of canopy closure and n is a crop dependent exponent, which is considered to be equal to 2.5 for lettuce [27].

The daily carbon gain (DCG, mol C m^−2^ d^−1^) is computed as follows:

DGC = 0.0036 H·α·PPFD(4)

where H is the photoperiod, α = CUE·A·CQY, and 0.0036 is a constant used to convert µmol to mol and hours to seconds.

The daily oxygen production (DOP, mol O_2_ m^−2^ d^−1^) is then given by a fraction (oxygen production fraction, OPF) of DGC:

DOP = OPF · DGC(5)

where OPF is expressed in mol O_2_ mol^−1^ C and is a crop-specific parameter.

The crop growth rate (CGR, g m^−2^ d^−1^) is given by:

CGR = MWc·DCG/BCF(6)

where MWc = 12 g mol^−1^ is the carbon molecular weight, while BCF is a crop specific parameter representing the biomass carbon fraction.

Thus, the total edible biomass (TEB), expressed as specific dry weight (g m^−2^), was calculated by integrating the crop growth rate, multiplied by the fraction of CGR allocated to edible biomass (XFRT):

(7) $$\mathrm{TEB}=\int_{\mathrm{tE}}^{\mathrm{tM}}\mathrm{XFRT}\;\cdot \;\mathrm{CGR}\;\cdot \;\mathrm{dt}$$

where t_M_ is the time of harvesting, t_E_ is the time of the onset of edible biomass formation and XFRT represents a partitioning coefficient for the edible biomass, which combines the effects of determinacy and temperature on storage organ growth rates [26]. 

The gross photosynthesis (P_G_, μmol CO_2_ m^−2^ s^−1^) is computed as follows:

P_G_ = β · PPFD (8)

where β = A·CQY and PPFD is the photosynthetic photon flux density (μmol m^−2^ s^−1^).

The net photosynthesis (P_N_, μmol CO_2_ m^−2^ s^−1^) is computed by accounting for the carbon use efficiency in the photoperiod:

P_N_ = [H·α/24 + β (24 − H)/24] · PPFD (9)

where H is the photoperiod, α = CUE·A·CQY, β = A·CQY and PPFD is the photosynthetic photon flux density (μmol m^−2^ s^−1^).

The stomatal conductance (g_S_, mol m^−2^ s^−1^) for planophile-type canopies (such as lettuce) is calculated according to Monje et al. [28]

g_S_ = (1.717·T − 19.96 − 10.54·VPD)·P_N_/[CO_2_] (10)

where T (°C) is the mean air temperature during light cycle and [CO_2_] is the air carbon dioxide concentration expressed as μmol CO_2_ mol^−1^.

The canopy surface conductance for water vapor (g_c_, mol m^−2^ s^−1^) is defined as follows:

g_c_ = g_A_·g_S_/(g_A_ + g_S_) (11)

where g_A_ = 2.5 mol m^−2^ s^−1^ is the aerodynamic conductance and g_s_ the stomatal conductance. 

The daily canopy transpiration (DTR, L m^−2^ d^−1^) is also calculated as follows:

DTR = 3600·H·(MW_W/_ρ_W_)·g_c_·(VPD/P_ATM_) (12)

where 3600 is a conversion constant from second to hours, MW_W_ = 18 g mol^−1^ is the molecular weight of water, ρ_W_ = 100 g L^−1^ is the water density, and P_ATM_ (kPa) is the total atmospheric pressure, which was used to convert vapor pressure to mole fraction.

### 2.2. Limitations of the Original MEC Model Formulation

**Figure 1:**
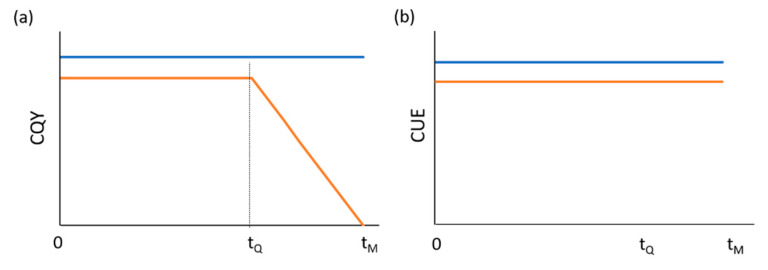
Original MEC model time profile of CQY (**a**) and CUE (**b**) for lettuce (blue lines), which was considered to be constant throughout the whole crop cycle; and for other crops (orange lines), in which CQY (**a**) was considered to be constant until the onset of senescence (t_Q_), to linearly decrease till the time of harvesting (t_M_), while CUE (**b**) was considered to be constant.

According to Equations (1)–(12), the original energy cascade model, used for advanced life support systems (ALSs) studies, predicted the biomass production and the photosynthetic rate based on three parameters: (i) the canopy light absorption (A), (ii) the crop quantum yield of PSII (CQY), and (iii) the carbon use efficiency (CUE). The physical and biological trends of these parameters were: a linear increase in PPFD absorption till the canopy closure; a constant CQY until the onset of senescence, followed by a linear decrease till the end of the cycle, and a constant CUE throughout the life cycle (Figure 1, orange lines). However, for lettuce and a few other crops like sweet potato, which are harvested before the occurrence of senescence (t_Q_), the CQY, as the CUE, were assumed to be constant during the entire growth cycle prior harvesting (Figure 1, blue lines). 

### 2.3. Experiments to Retrieve CQY Temporal Pattern

The canopy quantum yield (CQY) represents the moles of carbon fixed per mole of photons absorbed [29,30,31] and can be assessed in different ways such as:

(1) Dividing the daily P_G_ (mol C m^2^·d^–1^) by the total absorbed photons (mol m^2^·d^–1^) [32,33];
(2) From the initial slope of saturation-photosynthetic curves [29];
(3) By means of a fluorimeter, an instrument which measures the proportion of the light absorbed by the chlorophyll associated with the photosystem II (PSII), thus indicating the efficiency of the carbon fixation and of the overall photosynthesis [34].

In this study, we designed a calibration experiment for assessing the CQY temporal pattern in butterhead ‘Salanova’ lettuce (*Lactuca sativa* L. var. *capitata*), by means of a fluorimeter. The experiment was performed from 10 July 2019 to 6 August 2019, at the Department of Agricultural Sciences (University of Naples Federico II). Two weeks after sowing, the plants of butterhead lettuce cultivars, green- and red-leaf Salanova^®^ (Rijk Zwaan, Der Lier, The Netherlands) were transplanted into 10 cm pots filled with a peat:soil mixture (1:1 volume ratio) and exposed to solar light. Air temperature (T) was kept at 23 °C and plants were irrigated at 2-day intervals in order to reach the container capacity (till the beginning of drainage). Chlorophyll “a” fluorescence analyses were performed by means of a portable fluorimeter (ADC Bioscientific) on 3 expanded leaves per 4 green- and 4 red-lettuce plants every day, in order to highlight the leaf-age-driven variations in CQY, according to Genty et al. (1989) [35]. Fluorescence analyses were conducted at steady-state photosynthesis under a light intensity of about 400 µmol m^−2^ s^−1^, with a saturation pulse duration of 0.8 s, by keeping the orientation of the leaf relative to the actinic light source when taking CQY measurements. The leaves were chosen from three different positions within the lettuce head (top—t, medium—m, and bottom—b) in order to gain representative data for retrieving the general temporal pattern of CQY throughout the canopy.

### 2.4. Experiments in a Controlled Environment Growth Chamber to Validate the Model

**Figure 2:**
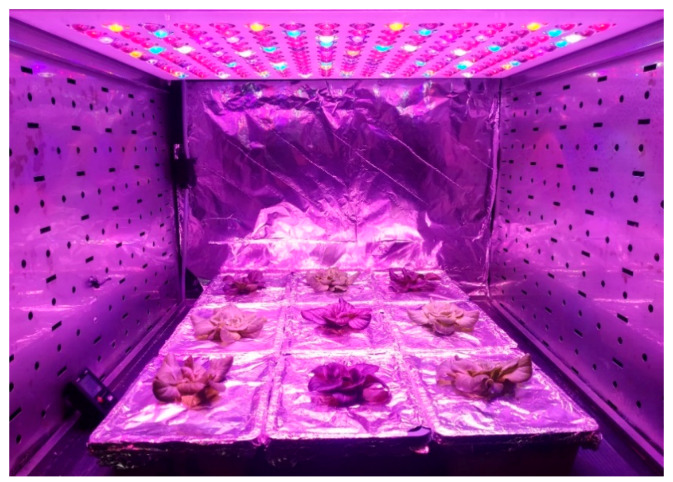
Climatic growth chamber with sensors used for the two cultivation trials (Low and High VPD) of green and red-leaf lettuce.

Nine green- and nine red-leaf Salanova lettuce plants were grown in a controlled environment growth chamber (KBP-6395F, Termaks, Bergen, Norvegia) (Figure 2), in two consecutive trials. In the first trial, 1-week old plants were transplanted into 10 cm pots filled with peat:perlite substrate (1:1 volume ratio) and incubated with an average VPD of 0.69 kPa, corresponding to nominal conditions of Low VPD. In the second trial, 9 green- and 9 red-leaf lettuce plants were incubated with an average VPD of 1.76 kPa, corresponding to off-nominal conditions of High VPD. The two different VPDs were achieved by keeping air temperature (T) constant at 24 °C, while changing the relative humidity (RH) accordingly. Temperature and RH were monitored and recorded every 10 min by means of mini sensors equipped with a data logger (Testo 174H). All other microclimate parameters and agricultural practices were the same in the two consecutive experiments. The lighting system was an RGB LED panel, with a light intensity of 315 PPFD µmol m^−2^ s^−1^ at the canopy level (12 h photoperiod; 13.6 daily light integral, DLI). Daily rotation of the trays was performed to ensure homogenous light and humidity across the shelf surface. Plants were daily weighted to assess the loss of water by transpiration (DTR) and were re-watered to field-capacity. Evaporation losses from the substrate were minimized by covering the substrate with a plastic film. Plant growth was assessed by imaging, measuring canopy total area every day, and counting the number of leaves. Furthermore, dry weight was recorded at the beginning and at the end of both trials. These measurements were used to reconstruct the daily total edible biomass (TEB). Changes in leaf temperature were monitored with an infrared thermometer on three leaves per plant (H-1020; Helect). These measurements were averaged and used instead of T in Equation (10) of the MEC model, to obtain more precise information of the leaf-to-air VPD which influences stomata conductance the most. After 23 days, on fully developed leaves, eco-physiological analyses in terms of gas exchanges (LCA 4; ADC BioScientific Ltd., Hoddesdon, UK) and chlorophyll “a” fluorescence (through the above-reported portable fluorimeter), were performed. Gas-exchange analyses were carried out on fully expanded leaves, using 9 replicates per condition to assess plants physiological behavior (P_N_ and g_s_) in response to different VPD conditions. During the measurements PAR, RH and carbon dioxide concentrations were set at ambient value and the flow rate of air was set to 400 mL s^−1^. P_N_ and g_s_ values were averaged and also used to evaluate the corresponding model prediction performances.

### 2.5. Model Structure and Parameter Identification 

The results of the experiments conducted to retrieve CQY temporal pattern suggested the opportunity to redesign the MEC model by modifying the temporal variability of some key parameters, without increasing the model complexity. Indeed, fundamental model outputs like TEB and P_N_, directly depend on these key parameters, which temporal patterns are influenced by the VPDs levels (nominal and off-nominal) and often result to be cultivar-specific [13]. Based on Equations (1)–(12), the three variables A, CQY, and CUE were reduced to the variable α = CUE·A·CQY and β = A·CQY. The developmental stages observed for CQY were then assumed to be valid also for α and β, i.e., under the assumption of functional similarity between the variables involved, as also suggested by other studies [36,37,38,39]. The key temporal parameters, explaining the different development for α and β, were set equal to those observed for CQY. The other parameters, identifying the minimum and maximum values for α and β, were calibrated by minimizing the root mean square error (RMSE) of the predicted DTR with respect to the corresponding observations over the entire simulation period, with the generalized reduced gradient optimization algorithm. The calibrated model was then validated against TEB measured over the entire simulation period. Simulated and modelled g_s_ and P_N_ were also compared on day 23 after transplanting (DAT), the day of the experiment when gas exchange analyses were performed to experimentally determine P_N_ and g_s_. Calibration and validation were performed for each of the four examined scenarios: green lettuce under nominal VPD conditions (G-N); green lettuce under off-nominal conditions (G-ON); red lettuce under nominal VPD conditions (R-N); red lettuce under off-nominal conditions (R-ON). 

The statistical performance indices for CQY, DTR and TEB were the linear correlation between predictions and observations (r), the average difference between prediction and observation (BIAS), the root mean square error (RMSE) and the ratio of performance to deviation (RPD). BIAS and RMSE were computed as follows:

(13) $$\mathrm{BIAS}=\frac{\sum_{j=1}^{N}{\left(X_{p,j}^{}-{\bar{X}}_{o,j}^{}\right)}^{}}{N}$$

(14) $$\mathrm{RMSE}=\sqrt{\frac{\sum_{j=1}^{N}{\left(X_{p,j}^{}-{\bar{X}}_{o,j}^{}\right)}^{2}}{N}}$$

where N is the number of simulation days considered for the computation of the performance index, $X_{p,j}^{}$ is the prediction on the j-th day, ${\bar{X}}_{o,j}^{}$ is the observation day on the j-th day, averaged among the 9 sample plants for each examined scenario. Meanwhile, the RPD was calculated as the ratio of the standard deviation of the experimental data to the standard error of the model predictions. Although considered redundant by some authors [40], it has been suggested that RPD > 3 are good for screening purpose, RPD > 5 are suitable for quality control and RPD values > 8 are considered optimal for every kind of analytical application [41]. The model performance for P_N_ and g_s_ were assessed by the relative BIAS, i.e., the BIAS normalized by the corresponding average measured value on the 23rd DAT.

## 3. Results 

### 3.1. Model Equations and Parameters

**Figure 3:**
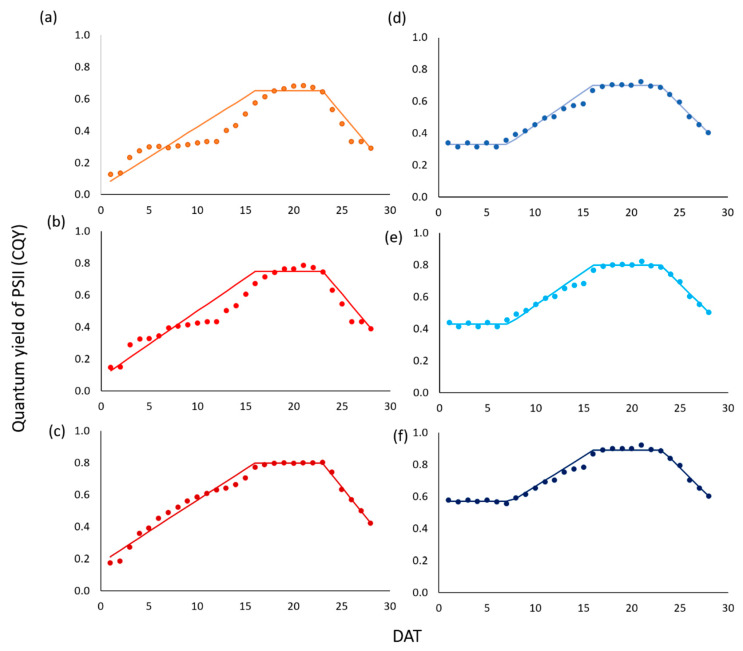
Profiles of CQY for top (**a**,**d**), medium (**b**,**e**) and bottom (**c**,**f**) leaves in the green- (**a**–**c**) and red-leaf (**d**–**f**) lettuce cultivars; model simulations (line) and experimental data (dots) are reported. Three different phases are identified: (1) a linear increasing; (2) a constant maturity; (3) a decreasing senescence plus an initial phase of stationary CQY for red-leaf plants. All data referred to 30 days after transplanting (DAT).

Results from fluorescence analyses on green- and red-leaf lettuce plants are reported in Figure 3. In both cultivars, top leaves always presented the highest values followed by medium leaves, while the lowest values were recorded in the bottom leaves. Based on the analyzed experimental data, in green-leaf plants, we distinguished three stages in CQY temporal patterns:

(1) A period of CQY monotonically increasing, starting from the initial leaf lamina development till the beginning of the maturity stage (t_Mi_).
(2) A period of stationary CQY, during plant maturity.
(3) A period of CQY monotonically decreasing, during senescence.

Furthermore, for the red-pigmented lettuce, the first phase was preceded by a period of stationary CQY.

In the light of these considerations, instead of Equation (1), we designed a new mathematical relation to analytically describe the temporal evolution of CQY, consistently with the experimental data. The first stage was modelled by a linear equation, that fits the observed data between the initial time of development (t_D_) and maturity (t_Mi_). The second stage was modelled by constant value, with CQY = CQY_MAX_, from t_Mi_ till end of maturity (t_M_). The third stage was modelled by a linear decreasing equation, between t_Mi_ and the time t_S_, corresponding to the end of the senescence period, with CQY = CQY_S_. The third stage is not relevant from a practical perspective, since it is beyond the time of harvesting, which coincides with t_M_. Moreover, for the red-leaf cultivar, the initial period of stationary CQY was modelled by a constant value, with CQY = CQY_MIN_, from the first day after transplanting till t_D_. This stage is only relevant for the red lettuce, with t_D_ = 8 days, since green lettuce does not present this “adaptation” period (i.e., t_D_ = 1). As illustrated in Figure 3, the other relevant times t_Mi_ and t_M_ resulted to be equal to 16 and 23 for all examined scenarios and leaves. 

As shown in Table 1, predicted CQY BIAS was close to zero, while RMSE varied from 0.028 to 0.072 for green- and 0.020–0.021 for red-leaf lettuce. However, the linear correlation between observed and predicted CQY was high in all conditions, always being larger than 0.92. Furthermore, the values for RPD always showed values around 5, indicating the robustness and reliability of CQY prediction model. 

In accordance with the assumptions stated in Section 2.5, variables α and β were modelled to change in time according to the following relation:

Χ = χ_MIN_   for t ≤ t_D_ χ = χ_MIN_ + (χ_MAX_ − χ_MIN_) (t − t_D_)/(t_Mi_ − t_D_) for t_D_ ≤ t < t_Mi_ χ = χ_MAX_   for t_Mi_ ≤ t < t_M_ (15)

where χ denotes the generic variable (either α or β), while χ_MIN_ and χ_MAX_ the corresponding minimum and maximum values. Parameters α_min_, α_max,_ β_min_ and β_max_ were derived from experimental gas-exchange and chlorophyll “a” fluorescence measurement performed in the growth chamber experiment. These parameters were therefore differentiated for the nominal and off-nominal scenarios and for green and red lettuce cultivars, since these cultivars showed different behaviors under the same growth conditions. Afterwards, these parameters were calibrated by minimizing the RMSE with respect to the measured DTR values. Table 2 presents the complete list of model parameters, including: parameters defined by the experimental setting (E), those set according to literature data (L1 and L2), those calibrated by means of the CQY experiments (C1), and those calibrated by means of the DTR measurements during the chamber growth experiments (C2).

### 3.2. Model Performance 

From our results, it is evident how DTR, TEB, g_S_, and P_N_ followed similar trends in green- and red-leaf lettuce cultivars grown under both nominal and off-nominal conditions, but with different absolute values and magnitude of changes during the cultivation cycles. 

**Figure 4:**
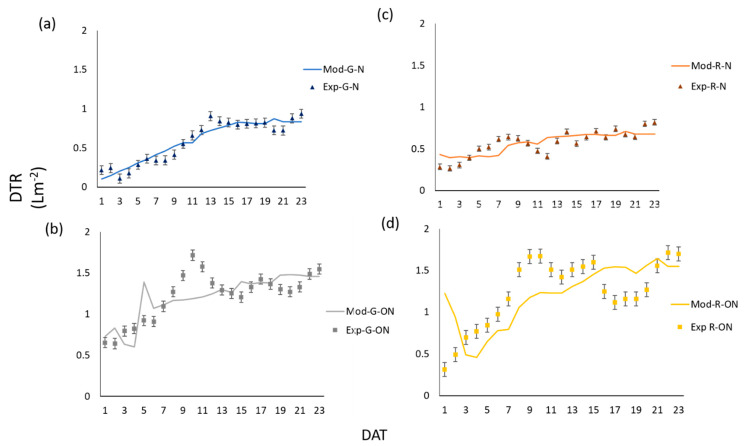
Profiles of DTR (Daily transpiration) for green- (G) (**a**,**b**) and red-leaf (R) (**c**,**d**) plants under nominal (N) (**a**,**c**) and off-nominal (ON) (**b**,**d**) scenarios; model simulations (line) and experimental data (dots) are reported.

Figure 4 shows the observed and predicted DTR values after model calibrations. The irregular pattern of DTR with time, is due to high sensitivity of DTR to slight perturbations in VPD levels during the diurnal hours of experiment. This sensitivity was higher under off-nominal conditions. Indeed, the model was able to reproduce the observed DTR under nominal conditions better than under off-nominal conditions. As reported in Table 3, under nominal conditions, predicted DTR BIAS was almost null, while RMSE was 0.09 L m^−2^ and 0.10 L m^−2^ for green- and red- leaf lettuce, respectively, i.e., less than 20% of the average DTR observed during the entire experiment. Under off-nominal condition, DTR BIAS was still low (max 0.04 L m^−2^ for red-leaf lettuce) but the RMSE increased to 0.21 L m^−2^ for the green lettuce and to 0.35 for the red lettuce. These RMSE values were still acceptable, since they are below the 30% of the observed DTR. The linear correlation between observed and predicted DTR was almost always high (larger than 0.70), except for the R-ON scenario which exhibited a linear correlation equal to 0.56. However, the RPD values varied from 5.10 to 6.40 for nominal conditions and 5.25 to 5.34 for off-nominal conditions. Being always higher than 5, the RPD overall indicates a good quality of the model predictions.

**Figure 5:**
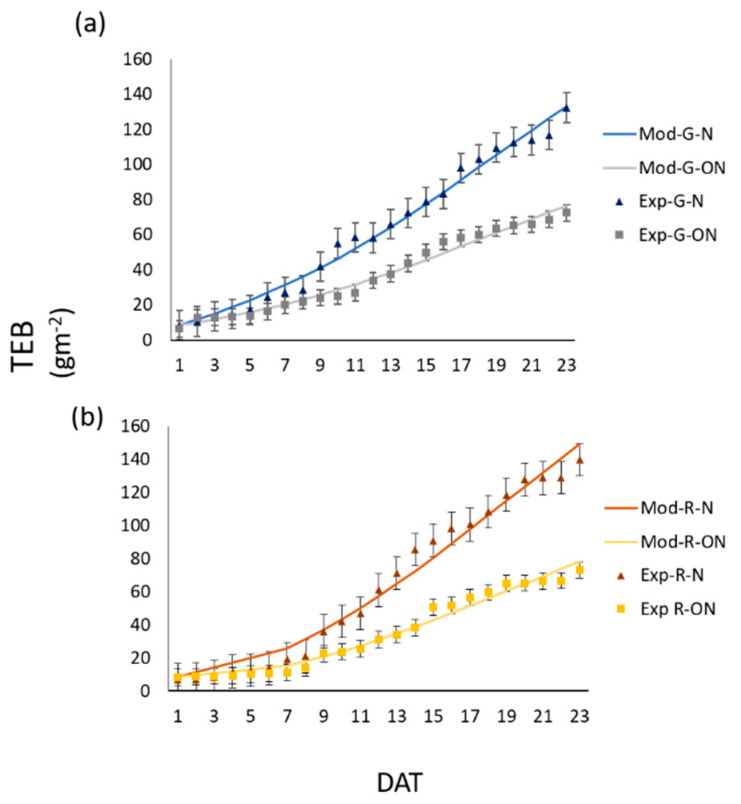
Theoretical (line) and experimental (dots) profiles of TEB (Total edible biomass) for green- (G) (**a**) and red-leaf (R) (**b**) plants under nominal (N) and off-nominal (ON) scenarios; model simulations (line) and experimental data (dots) are reported.

The calibrated model was validated with the observed total edible biomass (TEB), representative of the lettuce daily growth. Total edible biomass was also influenced by VPD conditions: plants under nominal condition developed more biomass than those grown under off-nominal scenarios, both in green- and red-leaf lettuce cultivars (Figure 5). The temporal evolution of TEB was more regular and less sensitive to the perturbations of the experimental settings. The TEB predictions curves fully reflected what was expected by lettuce grown under those different VPD conditions, showing an almost linear increment in biomass till the time of harvesting (Figure 5). Furthermore, the predicted growth curves accurately simulated the lettuce biomass accumulation. In Table 4, BIAS, RMSE, r and RPD for the TEB are reported. The linear correlation coefficient (r) was always close to 1, BIAS varied in the range 0.19–1.11 under nominal conditions, and 0.12–0.40 under off-nominal conditions, whereas RMSE and RPD values varied in the range 4.46–6.87 and 4.87–5.00 under nominal conditions and 2.98–3.60 and 4.88–4.96 under off-nominal conditions, indicating a good reliability of model predictions. Overall, the results showed that the TEB prediction errors were always below 10%.

**Figure 6:**
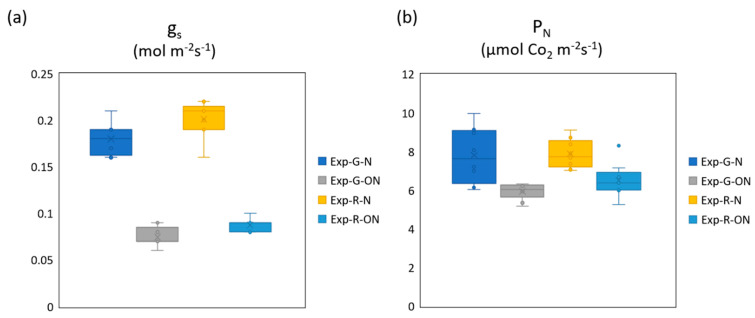
Box-plot distribution of g_s_ (Stomatal conductance) (**a**) and of P_N_ (Net-photosynthesis) (**b**) for green (G) and red (R) plants under nominal (N) and off-nominal (ON) scenarios. Experimental data referred to 23 DAT.

Figure 6 shows the box-plot distribution of the stomatal conductance (g_S_) as well as of the net photosynthesis (P_N_). Stomatal conductance and P_N_ were significantly higher under nominal conditions compared to off-nominal, in both butterhead cultivars. Unfortunately, these data are only available for the 23rd DAT, when leaf gas-exchange analyses were performed. As illustrated in Table 5, g_S_ relative BIAS (rBIAS) was equal to 39% and −0.1% in green- and red-leaf lettuce, respectively; P_N_ relative BIAS (rBIAS) was instead equal to 34.1% and −10.7%. Under off nominal conditions, the predicted g_S_ and P_N_ were much less accurate: rBIAS for g_S_ was 68.2% and 48.6% for green- and red-leaf lettuce respectively, while rBIAS for P_N_ was 75.9% and 70.9%. These larger overestimations of g_S_ and P_N_ testifies the limit of some empirical formulations (e.g., Equation (10)) adopted by the MEC model to reproduce the impact of off-nominal conditions on the stomatal conductance.

## 4. Discussion and Conclusions 

The proposed version of the MEC model, validated against experimental data on green- and red-leaf lettuce cultivars, grown under both nominal (low VPD) and off-nominal (high VPD) scenarios, proved to be reliable in predicting crop growth and transpiration rate. All previous versions of the MEC model considered CQY and CUE to be constant during plant growth [12,24,26,27]. This assumption for CQY and CUE constant behavior is not consistent with recent literature in which contrasting results on the topic are reported. For instance, in rice, Xu et al. (2019) [39] observed a decline in photosynthetic rate, dark respiration and quantum yield according to leaf aging. In the latter study, both parameters rapidly increased to a maximum around 15 days, to linearly decline as a response to plant aging. Similar findings were also reported in a study on *Rhododendron maximum* L., were the decline of CQY during leaf aging was exacerbated by the exposure to high light intensity [42]. 

The issue is even more complex for CUE, since the carbon use efficiency has been less characterized for horticultural plants and little information exists for lettuce under different environmental conditions [31]. Although many models still rely on a fixed value of CUE set around 0.5 [43,44], this topic has been questioned and more studies contrasting this theory have been reported. For example, Winzeler et al. (1976) [45] showed that CUE of barley increased during the early phases of the growing cycle, while a decrease was reported during the second half of the cultivation period. In forest species, Amthor (2000) [46] showed that CUE is reported to vary sharply with aging, within and among different species and environmental conditions, due to different respiratory needs for growth and maintenance [37,46]. Indeed, it should be noted that CUE represents how efficiently a plant incorporates carbon into biomass and can be defined as follows:

CUE = DCG/P_G_ = (P_N_ − R_D_)/(P_N_ + R_N_) (16)

where R_D_ and R_N_ are the daylight and night respiration, respectively.

Thus, a constant CUE would indicate that plants always present a constant positive respiration rate and that changes in photosynthetic activity would determine limited variations in growth and respiration, both these scenarios being quite unlikely [36]. Many studies have found that changes in the respiration rate during the plant growth cycle are species/cultivar-specific and maintain similar trends as net photosynthesis [38,39,47]. Furthermore, the situation can be different for the same plant species under different environmental conditions. Plant growth under near-optimal conditions have been reported to have smaller changes in CUE than plant grown under limited conditions, because the relative growth rate, higher under optimal conditions, would minimize the effect of maintenance respiration coefficients on the carbon use efficiency [36].

Given these uncertainties in the determination of CUE and the observed variability for CQY, in the present study, we suggested a modified version of the MEC model structure, by aggregating the variables A, CUE, CQY into two variables (α and β) and by assuming for these two variables the same temporal patterns observed for CQY till maturity, under the assumption of physiological similarity. Thus, the number of model parameters to be calibrated was reduced to four. The calibration was performed against the DTR data, while the remaining TEB g_s_ and P_N_ experimental data were used for validation. 

In the present study, we proved that in lettuce a temporal pattern exists in CQY changing during plant aging. Furthermore, our findings highlighted differences between green- and red-leaf lettuce plants. More specifically, a first stage of stationary CQY was observed just for the red ‘Salanova’ lettuce. This period could be attributed to the time required by this red-pigmented cultivar to adapt to the new environmental condition after transplanting. Indeed, green and red Salanova lettuce, although belonging to the same species, have been proven to have a different behavior even under the same environmental conditions. For instance, under both nominal and off-nominal conditions, red-leaf lettuce exhibited highest values of net photosynthesis, stomatal conductance, as well as a higher value of edible biomass [13]. 

Therefore, it was interesting to observe that all leaves of both green and red lettuce plants, notwithstanding the position inside the canopy level (top, medium, bottom), exhibited the same timing for CQY variation, except for the initial time of development (t_D_). The time t_D_ in red lettuce corresponds with the time of canopy closure (t_A_), which was also observed to be equal to eight days for both green and red Salanova. Thus, it is feasible that the red cultivar requires an initial time interval for adapting after the transplant. However, red plants completely recover from their initial “delay” by reaching CQY_MAX_ at time t_Mi_ = 16 days, as it occurs for green-leaf lettuces, also showing the highest values of net photosynthesis, stomatal conductance and edible biomass, overall suggesting a better physiological performance [48]. 

In our study, the experimental data and particularly TEB, g_s_ and P_N_ assumed the highest values under nominal condition, as a result of the lower evaporative demand. Under a high VPD (dry air), the evaporative demand increases, and plants try to counteract dehydration by closing their stomata, thus decreasing photosynthetic rates and stomatal conductance [5,49,50]. Indeed, under high VPD conditions, transpiration rates were enhanced, and a plant might lose water from tissues with negative consequences on the whole plant–hydraulic system. Thus, these plants require more water to reach the field-capacity, compared to those grown under nominal-conditions. Generally, the cultivation of crops under high VPD results in yield drop-off [51] and often in quality loss [52,53,54,55], which are considered major problems for crop production. 

This calibrated model was able to reproduce the observed transpiration and biomass growth, under both nominal and off-nominal conditions. This capability of prediction could represent an added value for the cultivation management in CEA because it may allow the prediction of any yield loss, consequent to sudden changes in the microenvironment. Furthermore, the reliability in the model prediction concerning the daily transpiration rates could allow the set-up of a precise irrigation schedule, according to changes in the environmental condition, similarly to what is done in other agricultural sectors, by developing decision support systems (DSS) based on the optimal combination of sensors and prediction models [56].

However, the model tended to overestimate stomatal conductance and photosynthesis under off-nominal conditions. A feasible technical explanation to these overestimations is that the empirical model was initially calibrated only for nominal conditions and that a “big leaf” approach is used to calibrate the model equations. A plant, especially when grown in a sub-optimal environment, triggers a cascade of biological processes leading to the development of leaves with different anatomical traits (especially those linked with conductance and hydraulics), thus influencing plant photosynthetic performance and the whole physiological behavior [57,58,59,60]. Therefore, in this specific case, overestimation of stomatal conductance and photosynthesis, while maintaining comparable values of transpiration under off-nominal conditions compared to nominal ones, can be explained by the lack of consideration of structural plasticity (e.g., mesophyll density and vein distribution) which can differentially establish the limits of different physiological processes [5]. In light of the above results, by applying this implemented version of the model to cultivation trials, it was possible to simulate variations in environmental parameters which can be due to sensor failure, power loss, and other problems related to environmental control. The present modified version of the MEC model can simulate crop growth, photosynthesis, and transpiration over a different range of environments and is therefore suitable to be implemented in decision support systems (DSS) for forecasting variations triggered by anomalies in the environmental control. However, the model still has a “big-leaf” approach and can therefore overestimate some processes happening at the crop morpho-physiological level. To increase the functionality of the model, a further step could be to modify the relation used to calculate g_s_ and P_N_ by considering morpho-physiological modifications that would affect plant gas exchanges under off-nominal conditions.

## Figures and Tables

**Table 1:** BIAS, root mean square error (RMSE), linear correlation (r) and ratio of performance to deviation (RPD) for CQY of PSII of green- (G) and red-leaf (R) lettuce cultivars for top-t, medium-m, and bottom-b leaves.

	BIAS	RMSE	r	RPD
G-t	−0.029	0.072	0.93	5.60
G-m	−0.027	0.065	0.96	5.61
G-b	−0.03	0.028	0.99	5.32
R-t	−0.05	0.021	0.99	5.44
R-m	−0.05	0.021	0.99	5.43
R-b	−0.05	0.020	0.99	5.23

**Table 2:** Parameters used to validate the modified MEC model.

Parameter	Definition	Value	Source
H	Photoperiod (hours)	12	E
PPFD	Photosynthetic photon flux	315	E
BCF	Biomass carbon fraction	0.4	L1
XFRT	Fraction of DCG allocated to edible biomass	0.95	L1
OPF	Oxygen production fraction (mol O_2_) (mol C)^−1^	1.08	L1
g_A_	Aerodynamic conductance for water vapor transfer	2.5	L2
t_D_	Red lettuce initial time of development (days)	8	C1
t_Mi_	Initial time of maturity (days)	16	C1
t_M_	Time of harvesting (days)	23	C1
α_min_	G-N	0.007	C2
	R-N	0.007	C2
	G-ON	0.003	C2
	R-ON	0.003	C2
α_max_	G-N	0.017	C2
	R-N	0.021	C2
	G-ON	0.010	C2
	R-ON	0.011	C2
β_min_	G-N	0	C2
	R-N	0.022	C2
	G-ON	0.049	C2
	R-ON	0.036	C2
β_max_	G-N	0.045	C2
	R-N	0.028	C2
	G-ON	0.056	C2
	R-ON	0.060	C2

In the source column: C1 = calibrated with CQY experiments as illustrated in Section 2.3, C2 = calibrated with chamber growth experiment as illustrated in Section 2.4; E = experimental setting, L1 from [15], and L2 from [13].

**Table 3:** BIAS, root mean square error (RMSE), linear correlation (r) and ratio of performance to deviation (RPD) for Daily Transpiration Rate (DTR) of green- (G) and red-leaf (R) lettuce cultivars grown under nominal (N) and off-nominal (ON) conditions.

	BIAS (L m^−2^)	RMSE (L m^−2^)	r	RPD
G-N	0.001	0.09	0.95	5.10
G-ON	−0.012	0.21	0.71	5.25
R-N	0.000	0.10	0.74	6.40
R-ON	−0.040	0.35	0.56	5.34

**Table 4:** BIAS, root mean square error (RMSE), linear correlation (r) and ratio of performance to deviation (RPD) for Total Edible Biomass (TEB) of green- (G) and red-leaf (R) lettuce cultivars grown under nominal (N) and off-nominal (ON) conditions.

	BIAS (g m^−2^)	RMSE (g m^−2^)	r	RPD
G-N	0.19	4.46	0.99	4.87
G-ON	−0.12	2.98	0.99	4.88
R-N	1.11	6.87	0.99	5.00
R-ON	0.40	3.60	0.98	4.96

**Table 5:** Relative BIAS (rBIAS) and model predictions of stomatal conductance (g_s_) and net photosynthesis (P_N_), for green- (G) and red-leaf (R) lettuce cultivars grown under nominal (N) and off-nominal (ON) conditions. All data are referred to 23 DAT.

	DAT	rBIAS g_s_	Predicted g_s_ (mol m^−2^ s^−1^)	rBIAS P_N_	Predicted P_N_ (µmol CO_2_ m^−2^ s^−1^)
G-N	23	39.4%	0.26	34.1%	9.80
G-ON	23	68.2%	0.13	75.9%	10.38
R-N	23	−0.1%	0.21	−10.7%	7.79
R-ON	23	48.6%	0.13	70.9%	11.12

## Abbreviations

ALSs	Advanced life support systems
A_MAX_	Maximum fraction of PPFD absorbed by the canopy
BCF	Biomass carbon fraction
BIAS	Average difference between prediction and observation
BLSSs	Bioregenerative life support systems
CEA	Controlled environment agriculture
CGR	Crop growth rate (g m^−2^ d^−1^)
CO_2_	Carbon dioxide (ppm)
CQY	Canopy quantum yield
CUE	Carbon use efficiency
DCG	Daily carbon gain (mol C m^−2^ d^−1^)
DLI	Daily light integral
DOP	Daily oxygen production (mol O_2_ m^−2^ d^−1^)
DTR	Daily canopy transpiration (mm d^−1^)
g_A_	Aerodynamic conductance (mol m^−2^ s^−1^)
g_c_	Canopy conductance (mol m^−2^ s^−1^)
g_s_	stomatal conductance (mol m^−2^ s^−1^)
H	Photoperiod
IoT	Internet of things
MEC	Energy cascade model
MWc	Carbon molecular weight (12 g mol^−1^)
MW_W_	Water molecular weight (18 g mol^−1^)
O_2_	Oxygen (ppm)
P_ATM_	Atmospheric pressure (kPa)
P_G_	Gross photosynthesis (μmol CO_2_ m^−2^ s^−1^)
P_N_	Net photosynthesis (μmol CO_2_ m^−2^ s^−1^)
PPFD	Photosynthetic photon flux density (µmol photon m^−2^ s^−1^)
PSII	Photosystem II
R_D_	Day respiration (μmol m^−2^ s^−1^)
RH	Relative humidity (%)
RMSE	Root mean square error
R_N_	Night respiration (μmol m^−2^ s^−1^)
SPAC	Soil-plant-atmosphere-continuum
T	Temperature (°C)
t_A_	Time at canopy closure
t_D_	Initial time of development
t_E_	Onset of edible biomass
TEB	Total edible biomass (g m^−2^)
t_M_	Time of harvesting
t_Mi_	Initial time of maturity
t_Q_	Time of the onset of senescence
T_S_	Time of senescence
VPD	Vapour pressure deficit (kPa)
XRTF	Partitioning coefficient for the edible biomass
ρ_W_	Water density (100 g L^−1^)
α	product of A, CQY and CUE
β	product of A and CQY
